# Urban Animals: Human-Poultry Relationships in Later Post-Medieval Belfast

**DOI:** 10.1007/s10761-016-0331-z

**Published:** 2016-04-22

**Authors:** B. Tyr Fothergill

**Affiliations:** School of Archaeology and Ancient History, University of Leicester, University Road, Leicester, LE1 7RH UK

**Keywords:** Animal husbandry, Urban, Women’s work, Poultry, Cock-fighting

## Abstract

Live animals were a ubiquitous feature of post-medieval cities and provided a variety of products to a broad cross-section of society. Poultry species were portable and accessible to people of modest means. Yet, the quotidian presence of poultry contrasts with the lack of attention to urban animal husbandry. Zooarchaeological data from the faunal assemblage from St. Anne’s Square, a 0.77 ha seventeenth to early twentieth-century site in Belfast, combined with historical legislation, court records, and news sheets held by the Public Record Office of Northern Ireland reveal the complexity of and contradictions implicit in poultry-human relationships in Belfast and nearby areas.

## Introduction and Research Background

Belfast was officially declared a city in 1888 after separation from County Antrim in 1865, and it was the largest city in Ireland by 1901. Despite the relatively short span of its cityhood in comparison with other cities in Britain and Ireland, Belfast has been the subject of an extensive archaeological review (Ó Baoill 2011) and many histories (Bardon 1982; Connolly 2012; Maguire 2009; Moss 1986; Owen 1921; Parkhill and Pollock 2010). A history of Belfast is beyond the scope of this work, but whilst published histories vary in terms of tone and perspective (later works tend to recognise a broader socio-economic spectrum and emphasise balance), they share a focus upon trade, industry, politics, and especially conflict. This pattern reflects the prominence and weight of these issues, which are deeply complex and intrinsic to modern identities. Beyond these themes, however, animals have shaped the urban experience both historically and archaeologically; they were of critical importance to the many-stranded economic success of Belfast. Yet, they and their roles have escaped investigation apart from bland acknowledgement as producers of waste, agents of disease, or shadowy features of the backdrop against which history happened. Certain species (namely pigs and poultry) were affordable to many city-dwellers and husbanded in these environments until well into the twentieth century, though urban pig-keeping was gradually legislated out of permissibility over time and few traces of poultry-rearing exist from *c*. 1920 onward. These “household animals” are easily overlooked and their maintenance often perceived as women’s work, which may have made them less intriguing subjects for historical writing (Thirsk 2006, p.262; Sayer 2013). Even economic histories of the keeping, movement, slaughter, processing, and sale of valuable and culturally important large ungulates such as horses and cattle remain obscure, heavily overshadowed by treatises on specific industries such as linen production and shipbuilding.

An archaeological perspective offers a distinctive way of examining husbandry methods and other past relations between humans and “household” domesticates in urban environments. Whilst brief interpretations based upon skeletal element measurements, species ratios, mortality profiles and population structure data collected at the time of primary analysis have clear utility with regard to understanding breeding, population management, and slaughtering practices, human perceptions of urban domesticates and other facets of their husbandry are not typically discussed from an archaeological perspective. These include subjects such as housing and space; transport and control of movement; waste management; grooming, hygiene, and medical treatment; and the harvesting, gathering, and sale of products. It is also important to recognise that different social groups in an urban environment will approach animal husbandry in ways which are not necessarily consistent or compatible with one another. Although the case of each species and city will be different, attempts to go beyond basic interpretations of animals raised in urban environments can offer enticing details of past relationships between humans and other animals beyond aspects of population and presence. In this paper, I examine the roles of poultry in Belfast by approaching the concept of husbandry as a suite of social practices that included a range of actors and interactions rather than a different way of framing exploitation or production. Poultry-human relationships can be detected in the histories of common pastimes, the urban economy, management of space, hygiene and sanitation, gender, and education; it is within these themes that aspects of husbandry emerge.

Zooarchaeological reports exist for a number of sites across Ulster and Northern Ireland (see Hamlin and Lynn 1988; Ó Baoill 2011), and although interpretations for the roles of large mammals, especially cattle and horses, are regularly provided, avian bone is not routinely discussed (or even analysed, in some cases). This is especially evident in reports from urban contexts in the post-medieval period, despite the fact that poultry husbandry would have been feasible in a far greater range of circumstances than horse or cattle husbandry. Furthermore, poultry-keeping had the potential to empower women across the socioeconomic spectrum and contribute to the financial stability of urban households and neighbourhoods during times of social upheaval. Several issues are responsible for the lack of archaeological attention; generally, these include perception, identification, and recovery. Post-medieval faunal assemblages are perceived as less valuable than those from other periods and are not prioritised for analysis or retention (Thomas 2009); identifying bird remains often requires specialist knowledge and comparative collections which are not easily accessible; and the diminutive size of avian (and small mammal) elements means that they can be easily missed unless small mesh sizes are used when sieving (O’Connor 2008, p.34). A lack of published faunal data from post-medieval sites in Northern Ireland is an additional barrier to research in this area.

**Fig. 1:**
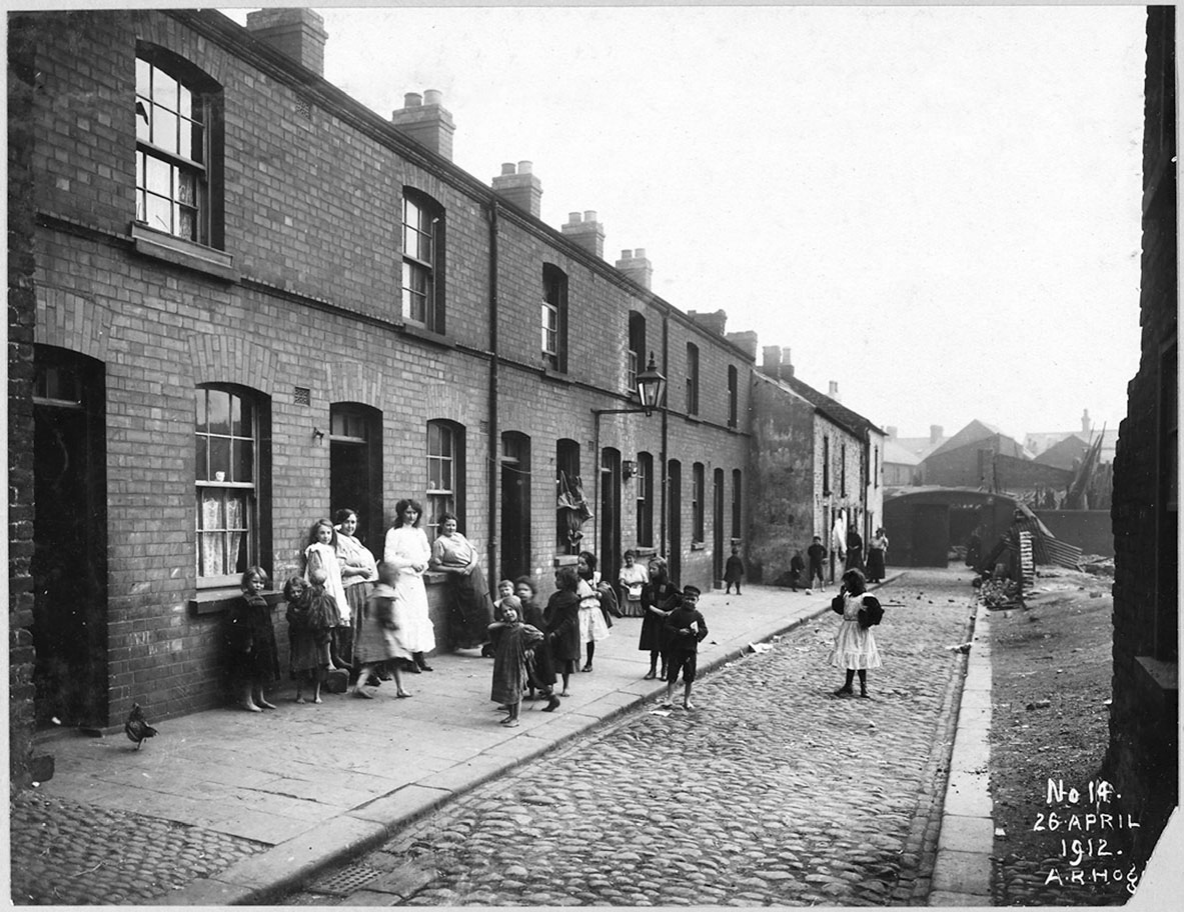
Abbey Street from Peter’s Hill toward North King Street with chicken in lower left corner (photo by Alexander R. Hogg) PRONI Ref: LA/7/8/HF/3/14

Poultry are also difficult to access through historical research in Britain and Ireland: 1.) as is the case with archaeological research, scholarly studies only infrequently focus on husbandry of avian species (though there are exceptions, e.g. Short’s article on chicken cramming in the Weald of Sussex (1982) and a portion of Thirsk’s book on alternative agriculture (1997)); 2.) women and their activities are practically invisible in source material as a by-product of statistical conventions and continuously-shifting categories of occupational recording (O’Hara 1994; Sayer 2013; Verdon 2009); and 3.) animal husbandry as an activity is only very exceptionally considered within the context of urban environments. The latter is somewhat surprising, given the evidence of its ancient origins and late continuations of such practices. Historical scholarship of animal husbandry has fixated upon rural management of large mammal species, mainly by men, and these associations have long echoes. When Philo (1995) engagingly approached the concept of nonhuman animals in nineteenth-century cities from a social geography perspective with an exclusive focus upon beings outside the realm of pets and commensals, poultry species were not included. Instead, the twin spectacles of slaughter-houses and meat markets (the former almost exclusively a masculine domain) were selected for examination. It may indeed be that the history of poultry husbandry received less attention from scholars due to an association with women, or that these animals and their products were valued less in past societies dominated by male convention, which meant that they were not considered worth documenting (Thirsk 2006, p.262; Sayer 2013). Anecdotal and photographic evidence certainly indicate that poultry were being kept in Belfast until at least 1912 (Fig. 1).

Within a mainly rural context, Joanna Bourke has described the value of chickens and their eggs as a source of power and independence in the lives of Irish women (1987; 1993), and Karen Sayer has produced a detailed qualitative analysis of the roles of women in British poultry-keeping from 1880 through a series of cultural transitions and societal upheavals into the post-war period (Sayer 2013). At certain points in the post-medieval period, chickens had other roles in male-dominated or exclusively male pastimes such as cockfighting, cock-throwing, and the breeding of fancy fowl, the histories of which have been discussed in works on a number of themes, including animal welfare, science, poetry, art, and sport (Atkinson 1891; Brewster and Reyes 2013; Cutter 1989; Donlon 1990; Forsyth 1978; Marie 2008; Secord 1981; Scott 1957; Strutt 1801). However, the husbandry of poultry in urban environments and the ways in which they and their products were perceived over time in that context have not been specifically addressed. Beyond this, deeper questions about the ways in which urban husbandry methods impacted both human and non-human animals and how these methods were altered by changing social attitudes to sanitation and emergent technologies warrant investigation.

The aims of this article are: to examine the poultry husbandry methods employed in Belfast and St. Anne’s Square more specifically, to identify the people who were keeping animals more broadly, to assess the skeletal and documentary evidence for urban poultry species, identify the diseases and injuries present in their remains, and determine whether changing attitudes to animals and hygiene impacted urban husbandry methods. Although post-medieval Belfast is the primary focus of this work, I also use faunal data from other sites of similar date in Ulster (Belfast 84, Dungiven Priory, and Bellaghy Bawn) and England (Plymouth, London) in order to highlight trends in skeletal metrics. Given the substantial limitations on both the archaeological and historical evidence for poultry-keeping, historical sources which refer to other parts of Ulster and the whole of Ireland are included when they inform upon practices in Belfast. This research highlights changing perceptions of these species and provides some groundwork for future exploration of relationships between human and non-human animals in Belfast and other cities in the post-medieval period.

## Materials and Methodology

### Zooarchaeology and Palaeopathology

The assemblage from St. Anne’s Square was excavated by NAC (Northern Archaeological Consultancy) in 2007 in preparation for a new retail development (Dunlop 2008). Standard zooarchaeological methods (see below) were used to record skeletal data from the remains of all species within the category “poultry” as it was conceptualised in much of the post-medieval period. This should include several avian species: pigeons, ducks, geese, chickens, and turkeys, but also rabbits, hares, and other smaller creatures. The deeply-intertwined histories of poultry and rabbit breeding societies may hearken back to this connection (Marie 2008). However, no rabbit or hare elements were identified during the St. Anne’s analysis and correspondingly, I have narrowed my approach to only avian members of the “poultry” category. Although I did not encounter them, rabbit bones were described in the original assemblage from Belfast 84, a site used for comparative investigations (below).

**Fig. 2:**
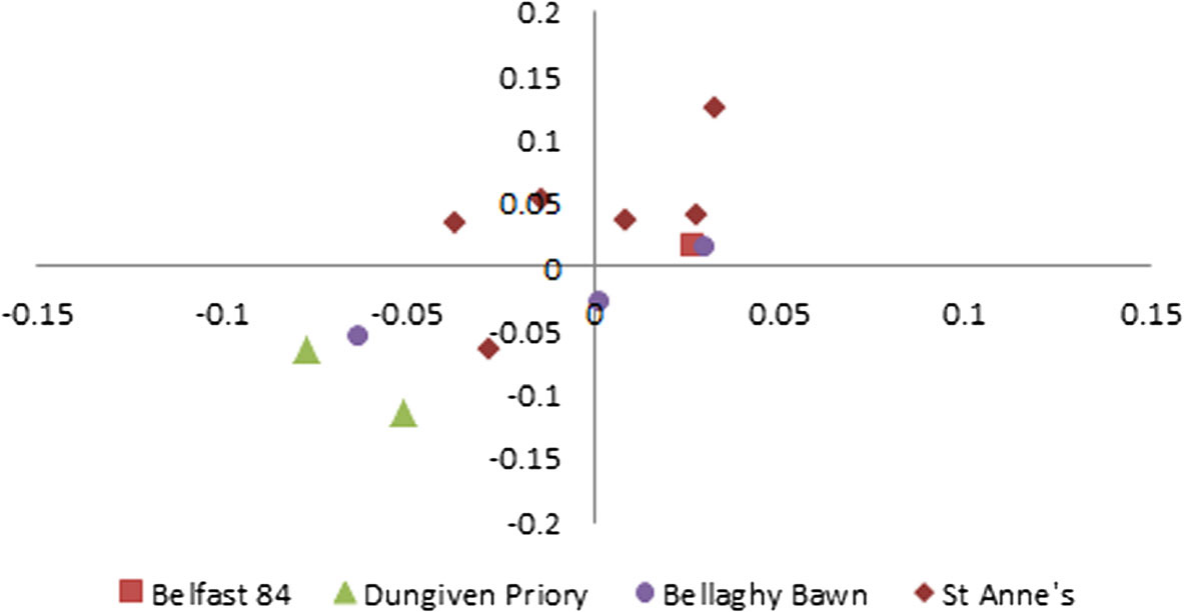
Log-scaled chicken element metrics from Belfast 84, Dungiven Priory, Bellaghy Bawn, and St. Anne’s Square

**Fig. 3:**
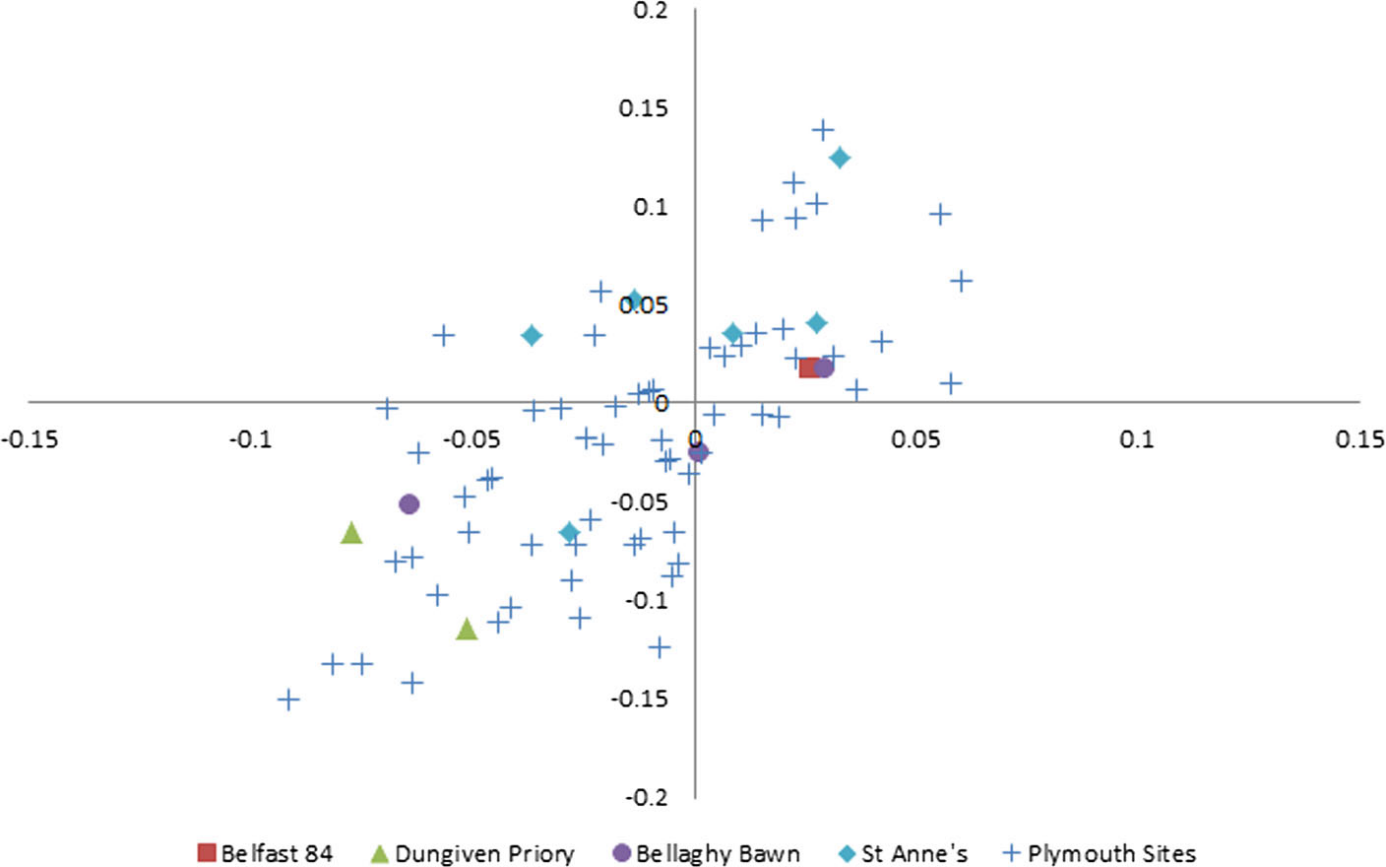
Log-scaled chicken element metrics from Belfast 84, Dungiven Priory, Bellaghy Bawn, St. Anne’s Square, and post-medieval sites in Plymouth

**Fig. 4:**
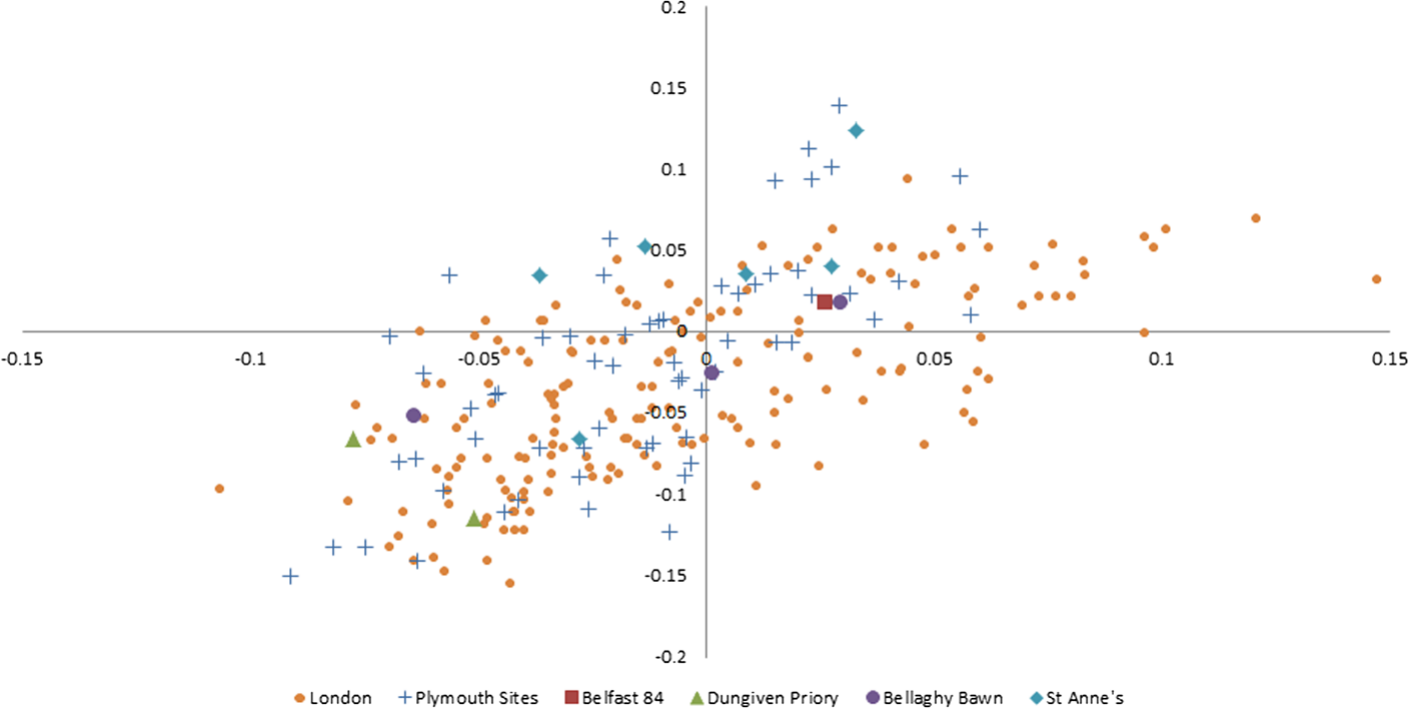
Log-scaled chicken element metrics from Belfast 84, Dungiven Priory, Bellaghy Bawn, St. Anne’s Square, Plymouth, and London

**Fig. 5:**
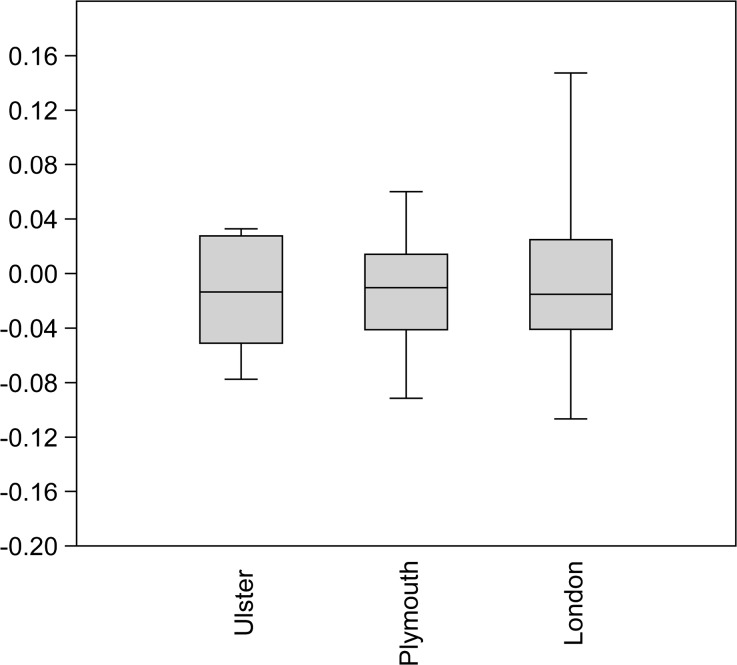
Mean averages and standard deviations of log-scaled chicken element lengths from the Ulster sites, Plymouth, and London

**Fig. 6:**
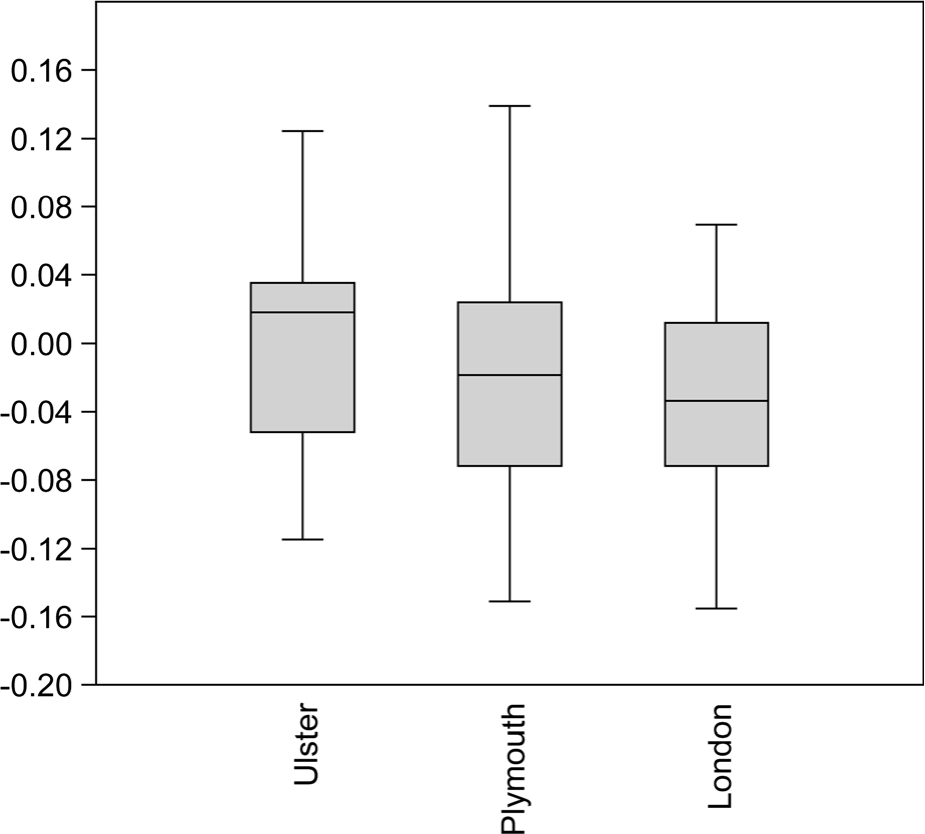
Mean averages and standard deviations of log-scaled chicken element breadths from the Ulster sites, Plymouth, and London

In addition to the material from St. Anne’s Square held at NAC in Belfast (Denham 2008), I analysed avian remains from other assemblages of post-medieval date which had been retained at the Northern Irish Environment Agency depot in Moira. These analyses were intended to complement the evidence from St. Anne’s Square with regard to the linear measurements of avian skeletal material (Figs. 2, 3, 4, 5 and 6). These were: Belfast 84, a group of late seventeenth-century gardens and properties at Pottinger’s Entry in Belfast (Brannon 1988, p.79–81); Dungiven Priory, a secular seventeenth-century re-occupation of a monastic site (Brannon 1985, 1988, p.81–84); and Bellaghy Bawn, a seventeenth-century fortified castle near a planter village which now houses the Seamus Heaney Centre (Brannon 1989; Horning 2006).

There are limitations on the evidence presented here. The excavated assemblage from St. Anne’s Square was not complete at the time of my analysis. Although the original faunal analysis was conducted on five numbered crates of bone (Denham 2008), only three of these were present in the store by the time of my arrival at NAC in 2013, and the location of the other two crates was not known. Furthermore, the majority of avian bone was excavated from Context 1, the uppermost archaeological stratum across the entirety of the site, which was comprised of sand, clay, brick and slate rubble (Dunlop 2008, Appendix 2). A more refined spatial extent (e.g. to the level of street or house) is thus unfortunately not available and the avian remains cannot therefore be linked to specific dwellings. Other contexts have three avian fragments at most, and are mainly ditch, drain, or pit fills (Contexts 4, 107, 159, 239, 241) with the exception of context 728, the wall foundation for the west wall of the Young, King, & Co. building at the corner of Talbot Street and Robert Street (Dunlop 2008, 119, Appendix 2), which contained a partial goose humerus.

All accessible avian bone fragments were measured, sided, and zoned, regardless of pathology, using Cohen and Serjeantson’s methodology (1996, p.109–112); measurements were taken to a tenth of a millimetre. I also collected zooarchaeological data on age and sex, butchery type and location, rodent and carnivore gnawing, burning, and root etching. Identification of juvenile elements was contingent upon a porous appearance; some very young elements could be identified only to order. Juvenile bones were not sexed. Metrical clustering analysis could not be used to sex elements due to the small size of the sample. No spurred tarsometatarsi were identified, but medullary bone was detectable in two elements. Pathologies were described using the protocols established by Vann and Thomas (2006), and examined both macroscopically and microscopically before they were photographed.

### Archival Research

On the premise that different information would be revealed by each type of archival document, I examined as many varieties of these as possible. Each kind of document required a slightly different approach, primarily due to variation in accessibility, formatting, and legibility. Local Acts, bylaws, council records, and other documents issued by the Crown were easily accessible in paper format and could be quickly reviewed for pertinent data, but variable handwriting styles and issues of preservation made some of the earlier court records very difficult to read. Newspapers were preserved only on reel microfiche, and hence more time-consuming to examine. The varying time spans required to interrogate each document type meant that it was necessary to adopt a strategy of targeting only specific years of court records and microfiche material, whereas the available legislation pertinent to animals, the spaces in which they were permitted, their husbandry, slaughter, and marketing, etc. could be studied in their entirety. Court records in which Belfast was included were reviewed from 1822 onward, yearly at decade intervals where extant: the Crown Book at Quarter Session for the County of Antrim for 1825 and 1847; the Crown Book at Quarter Session for Carrickfergus for 1855–1895; and the Crown Book at Quarter Session for Belfast for the years 1904–5. With regard to newspapers, two periodicals were chosen in an attempt to keep perspectives balanced and also to cover as broad a timeframe as possible: the Belfast Newsletter and the Belfast Morning News. The Newsletter was viewed as a unionist paper, and the Morning News as broadly nationalist (Bartlett 2014). Every issue of each year chosen was visually scanned in order to pull out seasonal trends. The earliest newspapers available on microfiche were from the Belfast Newsletter; I examined all issues from 1738 to 1749 on the basis that I had fewer sources from earlier periods and read entire years at two-decade intervals from 1765 to 1845. For the Belfast Morning News, I read entire years at one-decade intervals from 1859 to 1879. I also read the complete Minute Books of the Public Health Committee of Belfast from 1887 to 1896, 1896–1899, and 1906–1908.

## Results

### Zooarchaeology and Palaeopathology

The analysed portion of the St. Anne’s Square assemblage contained only 37 bird bones (6 % of the total number of identified specimens; Table 1), but it is likely that the two missing boxes would increase this number. Furthermore, the usual problems of preservation which differentially affect avian elements should be considered. The mammal assemblage is also not large: the site report provides a NISP of 563 without cattle horncores (Denham 2008).

**Table 1:** Taxa identified in the St. Anne’s Square faunal assemblage

	Taxon	NISP
Aves	Unidentified avian	5
	Anseriformes	1
	*Anas sp*.	1
	*Anser anser*	5
	Galliformes	4
	*Gallus gallus*	18
	*Meleagris gallopavo*	2
	Corvidae	1
Total Aves		37
Mammalia	*Rattus sp*.	1
	*Canis l. familiaris*	8
	*Meles meles*	1
	*Felis catus*	4
	*Equus f. caballus*	129
	*Sus scrofa*	55
	Caprine	58
	*Bos taurus*	307
Total Mammalia		563
Total		600

On the supposition that the species proportions present in the assemblage are roughly consistent in the material which could not be analysed, avian elements may originally have numbered approximately 60, which would then make the avian percentage of total NISP closer to 10 %. In any case, an avian proportion of 6–10 % is consistent with the accessibility of poultry species, suitability of domestic avians for urban husbandry strategies, and the persistence of poultry (especially chickens) in some anecdotal and very few documentary sources. Butchery was present on 12 bones and other modifications were evident: 11 of the avian bones were gnawed by carnivores, and 5 of them by rodents (which might indicate surface exposure of elements before disposal, or a series of depositional events). One goose and one unidentified avian element bore evidence of burning. A combination of highly variable preservation and small sample size prevented further investigation of patterning in bone modifications. Although the avian skeletal assemblage from St. Anne’s Square was not large (Table 1), it included six juvenile elements. One of these was so young as to defy taxonomic identification more specific than galliform, though in this context, it is most probably a chicken. It is therefore likely that this bird, and perhaps the other juveniles, were bred in Belfast. Also, the presence of medullary bone indicates that two chickens were in lay at the time of their deaths.

In the St. Anne’s Square assemblage, only the chicken remains returned multiple measurements for both element length (GL) and breadth (SC; Bb for coracoids; Bp for carpometacarpi). By log-scaling these measurements against the corresponding value from the same skeletal element in a set of standard chicken measurements, multiple different elements can be plotted on the same graph and compared to other assemblages. The metrics used as log-scaling standards in Figs. 2, 3 and 4 are from a modern dual-purpose Warren-Ranger hybrid hen held in the comparative collection at the University of Leicester (also used in Thomas et al. 2013). Figure 2 shows the log-scaled values for these elements from St. Anne’s Square, along with those from chicken bones from the Belfast 84 excavation, Dungiven Priory, and Bellaghy Bawn. The log-scaled measurements from the latter three sites are provided to contextualise the St. Anne’s measurements with other data from post-medieval sites in Ulster.

Firm comparative conclusions about this distribution are not possible because these samples are small and unlikely to be representative (and chickens are sexually dimorphic), and metrical clustering is weak due to the small assemblage sizes. At best, it may suggest that a majority of the chicken elements from these sites were from hens.

A comparison with chicken element measurements from other areas is somewhat more revealing. As part of a different project, I collected metrics from chicken bones in assemblages from post-medieval sites in Plymouth which were excavated by the Plymouth City Museum, the Exeter Museums Archaeological Field Unit, or Exeter Archaeology: the Kitto Institute (Allan and Barber 1992), PA76, Dung Quay (PDQ01; Stead 2003), PPD06, PP7 96, and Vauxhall Street (PVS90; Fig. 3). When these were log-scaled together with the data from St. Anne’s, Belfast 84, Dungiven Priory, and Bellaghy Bawn they aligned reasonably well and clusters which may represent sexual dimorphism became visible (hens in the large group on the lower left and cocks in the small group, upper right).

However, when the data from St. Anne’s, Belfast 84, Dungiven Priory, Bellaghy Bawn, and Plymouth are compared to the metrics from post-medieval London (Thomas et al. 2013; Fig. 4), a different pattern emerges. If taken as a whole, the log-scaled values from sites outside of London are spread out along a steeper slope.

Figures 5 and 6 below provide a different way of comparing these data. They show the minimum and maximum values, interquartile ranges and medians for the log-scaled lengths and breadths from St. Anne’s, Belfast 84, Dungiven Priory, and Bellaghy Bawn (in the “Ulster” group), sites in Plymouth, and sites in London. London has the largest values with regard to element length, and also the greatest range of lengths in comparison to the other two data sets. Whilst London also has the smallest breadths, some breadth measurements from sites in Ulster and Plymouth exceed the values from London chickens.

**Fig. 7:**
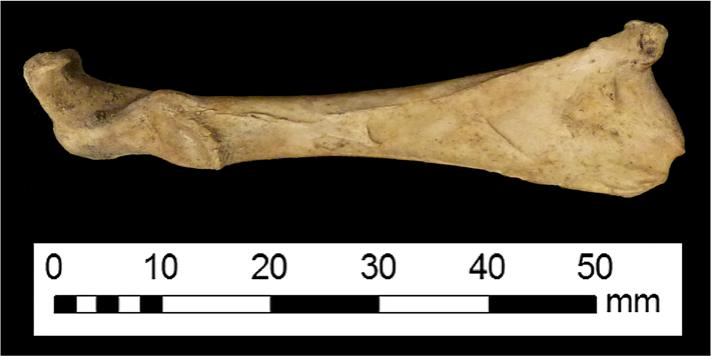
Coracoid with lesions consistent with age-related arthropathy

**Fig. 8:**
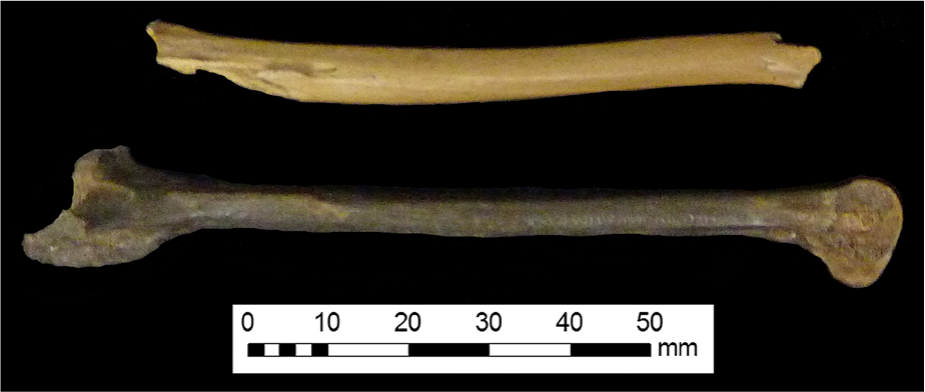
Tibiotarsus fragment with slightly warped appearance

Pathology is present in two elements from the St. Anne’s Square assemblage (Figs. 7 and 8). One coracoid exhibits lesions which are consistent with age-related joint disease, and a fragmentary tibiotarsus shows slight bowing.

### Cockfighting and Cock-Throwing

**Fig. 9:**
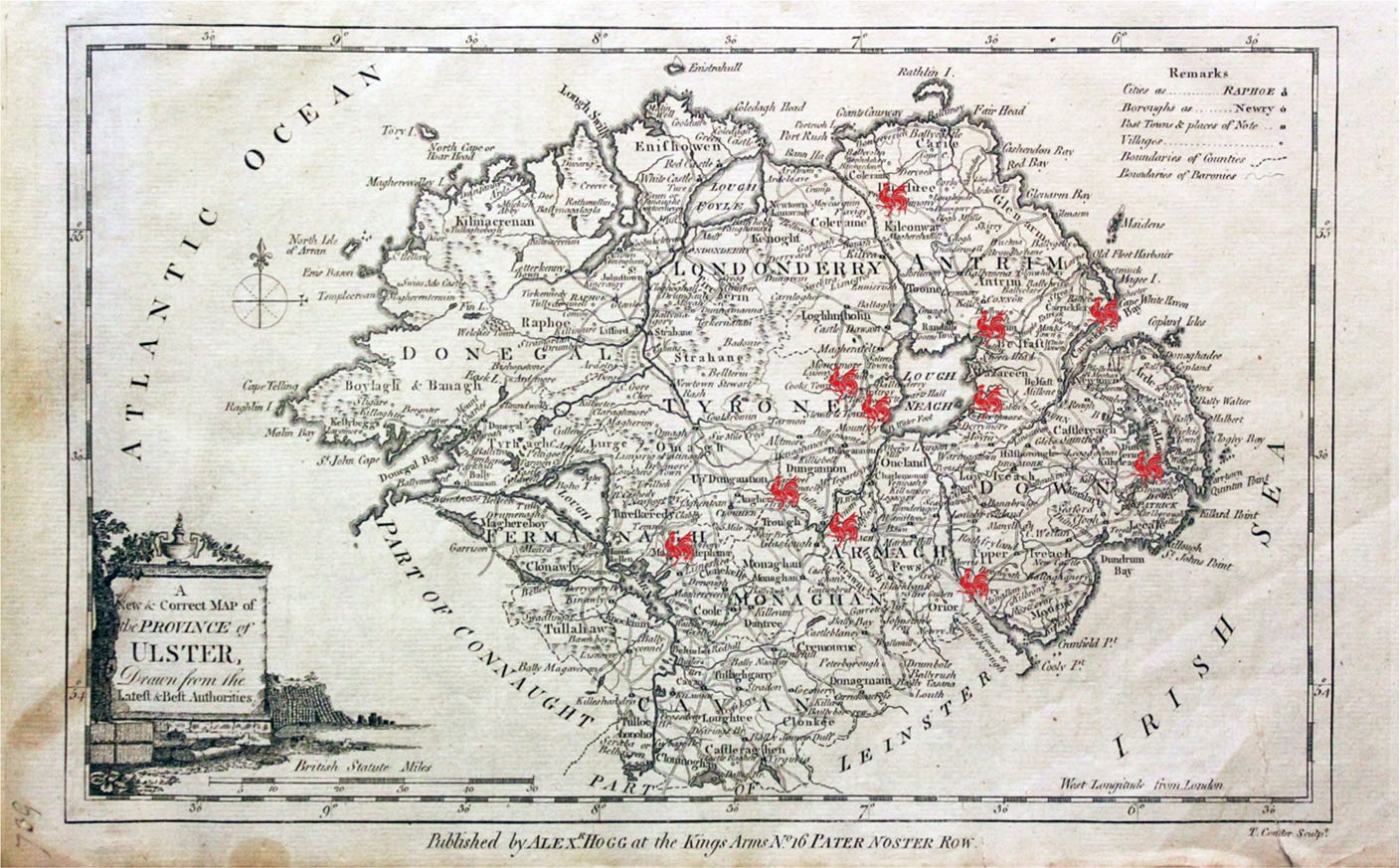
Eighteenth-century cockfighting event locations mapped onto Conder and Hogg’s 1780 map of Ulster

The earliest mentions of poultry in the archival sources were printed in the Belfast Newsletter and concern cockfighting. Cockfighting was a kingly sport for a time in many places, and Britain and Ireland were no exception (the cockpit built by Henry VIII at Whitehall was replaced by the Privy Council room after a fire in 1697 (Strutt 1801, p.224)). Cockfighting became a more prevalent diversion over time and both cockfighting and cock-throwing had evolved into a public spectacle by the time they were completely banned by the Act for the Improvement for the Borough of Belfast in 1845 (nearly a century after cock-throwing featured in the first of William Hogarth’s 1751 series of engravings: *The Four Stages of Cruelty*). These pastimes were, however, viewed as a nuisance long before they were proscribed by the Crown: in 1749, a notice was given in the Belfast Newsletter that both cockfighting and cock-throwing were forbidden in Belfast and within a two-mile radius of the town on Shrove Tuesday (Friday, February 23rd), and disturbances due to cock-fighting were amongst the regular issues which the nineteenth-century Belfast police force had to contend with (Griffin 1997, p.106). After the Shrove Tuesday ban, other mentions from the eighteenth century in the Belfast Newsletter concern areas outside of Belfast. From the examined records, cockfighting appears to have been a summer pastime, with advertised events taking place from early May to mid-August in both 1765 and 1785. Ascension Day in particular was viewed as an auspicious day for sport in Ireland, including cockfighting (Collins et al. 2005, p.57). Indeed, on Ascension Day of 1765 (May 16th), a cockfighting and horse-racing event was held at Carrickfergus, attended by gentlemen of Carrickfergus as well as the counties of Down and Antrim (Belfast Newsletter, 14 May, 1765a). The dates of cockfights did not overlap with each other over the course of a season. Events included fights between “stags” (young cocks under 11 months of age), “cocks” (experienced adult fighting cocks), or both; attendees could wager on the outcomes of individual battles or a series thereof. Cockfighting events described in the Belfast Newsletter lasted from 3 to 6 days, with the birds being shown, weighed and described on the first day, which resembles the structure adopted for English cockfights (Sketchley 1814, p.31, p.59). Figure 9 below shows the locations of cockfights as mentioned in the eighteenth-century issues of the Belfast Newsletter on a 1780 map of Ulster (Conder and Hogg 1780). There are some changes over time: the locations used in 1765 are not advertised for cockfighting in 1785, and the 1765 events are held at Ballymoney, Antrim, Carrickfergus and Newry, with later events taking place further west and south.

On Friday, June 14th and Tuesday, June 25th of 1765, an advert for the Ballymoney Races on the twelfth to seventeenth of August ended with the statement:

“A considerable Main of Cock-fighting on the Morning of each Day, and a Ball each Night. Distinct Ordinaries for Ladies and Gentlemen.” (Belfast Newsletter 14 June, 1765c and 25 June, 1765d)

Although it may seem unusual that ladies are mentioned at all in the context of cockfighting events since it is conventionally viewed as a masculine activity, women in England attended cockfights until the mid-nineteenth century and some even trained their own fighting cocks (Collins et al. 2005, p.72). The majority of notices for cockfights are formulaic and list the date, location, number of stags and cocks to be involved (usually 15 or 31), and the counties from which the gentlemen involved originated. Half of 1765 adverts include horse-racing, whilst those from 1785 do not (nor do they mention women or balls):

“Cock-Fighting. THE second of May next a Cock-Main to be fought at Maguire’s-bridge, between the Gentlemen of the County of Armagh and the Gentlemen of Fermanagh, for four Guineas a battle, and one hundred the odd one: Also a Stag-Main between the same parties, to be fought at Aughnacloy 27th June next, for the like sum. April 16th, 1785.” (Belfast Newsletter, 15–19 April, 1785a)

Betting on a single battle cost around four guineas on average, but betting on the entire main or “odd one” varied from 20 guineas (Belfast Newsletter, May 27–31, 1785c) to two hundred guineas (Belfast Newsletter, May 28, 1765b). The term “odd one” refers to the practice of using odd numbers of cocks or stags for each event, a system employed to prevent draws (Atkinson 1891, p.49). The vast majority of cockfights advertised in the Belfast Newsletter were of this type, and I found only a single instance of a different variety of main: a 16-bird cockfight at Stewartstown from the 4th of June onward (Belfast Newsletter, 24–27 May, 1785b). This was probably a “Welsh main”, which used 8 or 16 birds in an increasingly brutal knock-out system somewhat resembling the Rugby World Cup: by the time a single bird won, it had been fought at least four times (Collins et al. 2005, p.71). Despite the fact that cockfighting was not banned until four decades later, all mentions of cockfighting (and thus poultry species altogether) disappeared from the pages of the Belfast Newsletter by 1805. This is not to say it did not continue; cockfighting and other blood sports were “attended by gentlemen of the town as well as by poorer people” in the first half of the nineteenth century, with multiple cock-pits operating within and just outside of Belfast (Griffin 1997, p.104–105). Griffin does point out that the wealthier members of society rapidly lost interest in the pastime following the ban in 1845 (1997, p.106). He quotes the September 1847 issue of the *Belfast People’s Magazine*, wherein cockfighting is described as “almost exclusively confined to the dregs of society” (Griffin 1997, p.105).

### Theft and Sale

Once cockfighting was no longer advertised, aspects of poultry species are less prominent in the archival source material, but court records mentioning poultry theft do begin to crop up from the early 19th century. Court records from post-medieval London indicate that poultry, especially geese and turkeys, were a frequent target for theft (Fothergill 2014, p.212–215), particularly at Christmastime, and there is some evidence for a similar trend in the records from County Antrim. The Crown Book at Quarter Session for Country Antrim recorded that on the 24th of December, 1824, Rose Connell stole a goose worth 6 pence that belonged to John McIlreavy of Boston. The Antrim records also show that in 1847, James Adams stole Richard Casey’s goose at Ballypostry on the 10th of March. The Belfast Crown Book at Quarter Session has an entry for the 23rd of December in 1904 which records that George Beggs stole both a goose and a turkey, but the name and address of the owner of these birds were not readable.

**Fig. 10:**
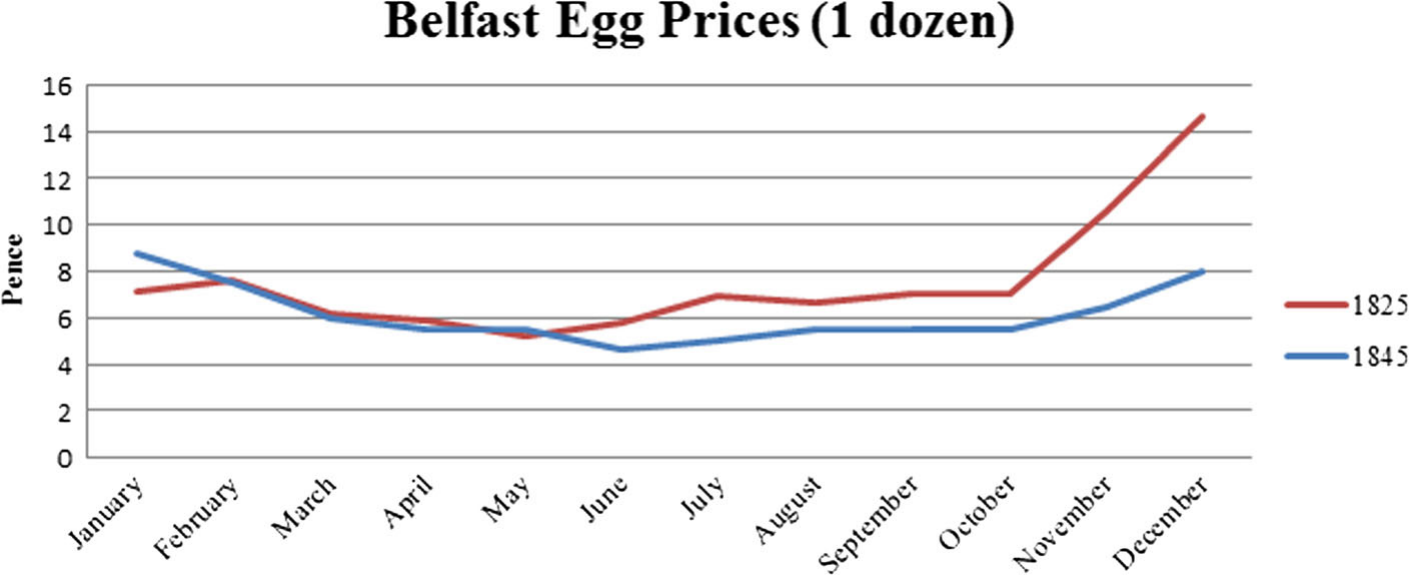
Prices for a dozen eggs in Belfast for the years 1825 and 1845

Market prices for hen’s eggs were advertised in the Belfast Newsletter by 1825 and this continued in 1845a (Fig. 10). Chickens lay more prolifically during the spring and summer months, and it is evident from the near-doubling of egg prices during the winter months that seasonal laying must have continued to some extent, though this appears to become less of an issue during the intervening decades and the price increase is less severe.

The lower egg prices in spring coincided with increased hours of daylight (and therefore improved egg production) as well as the seasonal Easter fairs at which “egg-trundling” took place. In writing about Holywood, County Down in 1819, William Shaw Mason described the pastime, which he links exclusively to Presbyterians, near Belfast:

“The trundling of eggs, as it is called, is another amusement, which is common at Easter. For this purpose the eggs are boiled hard, and dyed of different colours, and when they are thus prepared, the sport consists in throwing or trundling them along the ground, especially down a declivity, and gathering up the broken fragments to eat them. Formerly it was usual with the women and children to collect in large bodies for this purpose, though nothing can be, to all appearance, more unmeaning than the amusement; and they yet pursue it in the vicinity of Belfast.”(Mason 1819, p.14)

**Fig. 11:**
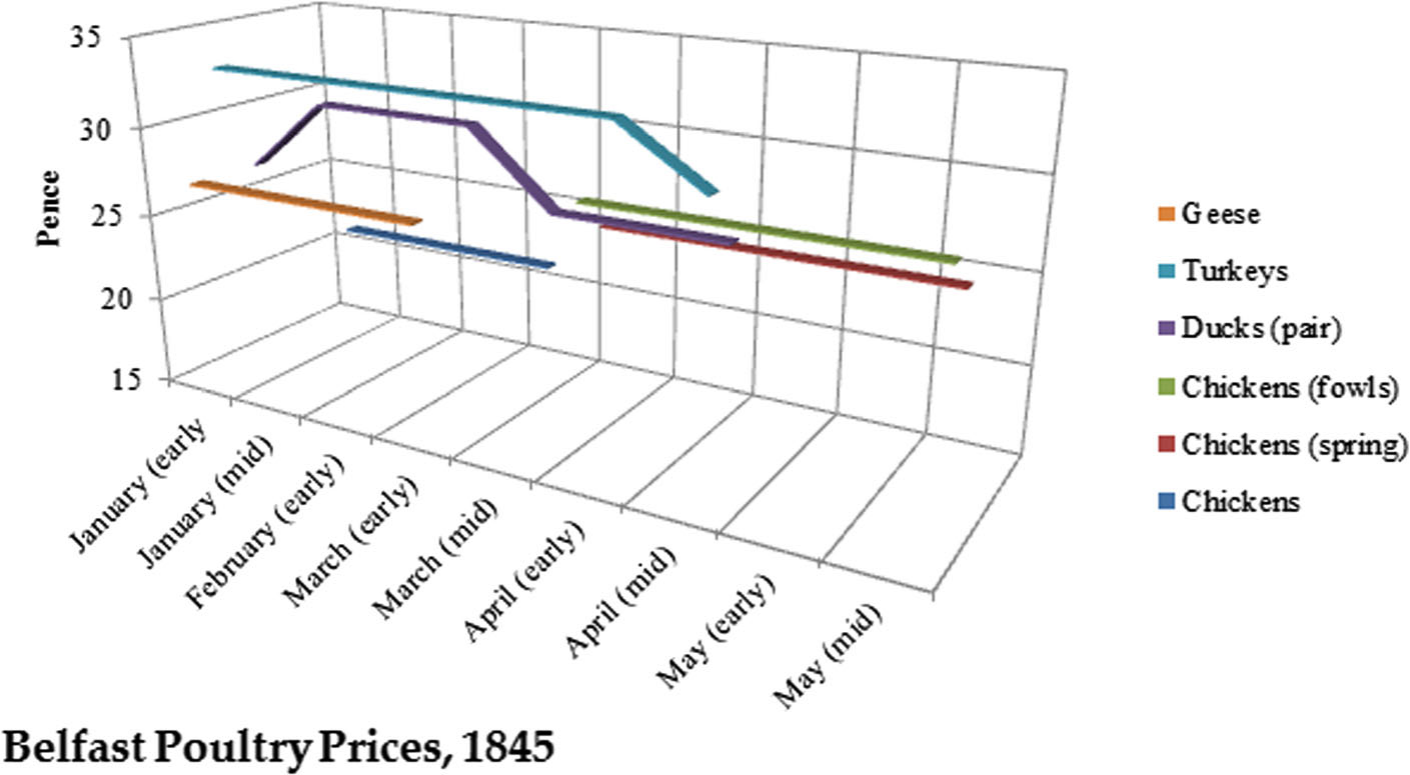
Prices for poultry in Belfast markets for the first half of 1845

The pricing data I obtained on poultry is limited to the first half of 1845, but even so, the extreme seasonal fluctuations that affect hen’s eggs are not evident. In mid-March, these categories change from “Chickens” to both “Chickens (spring)” and “Chickens (fowl)”, with the latter fetching a higher price (Fig. 11). The absence of turkeys and geese after March correlates neatly with Isabella Beeton’s description of seasonal market availability for these species in England, though to what extent Belfast should resemble this is not clear (1861, p.50–55).

**Fig. 12:**
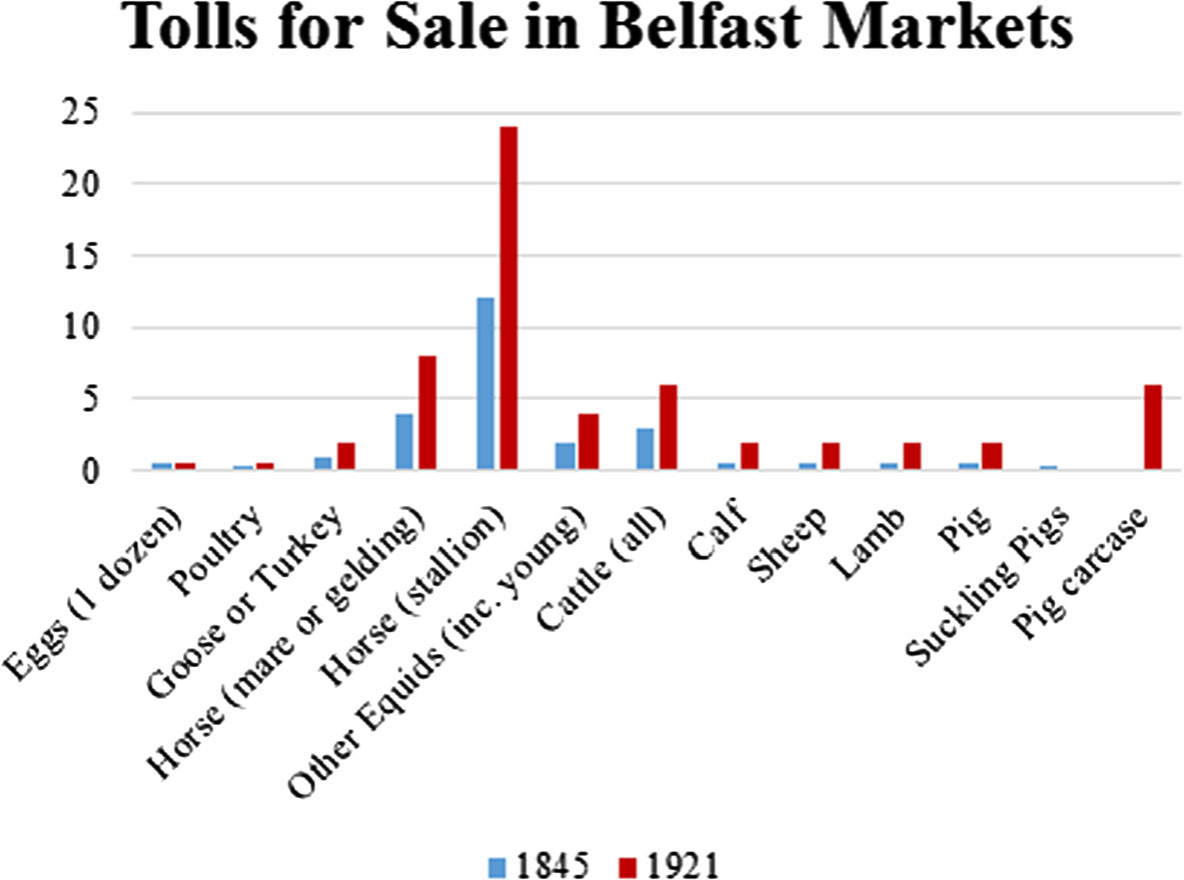
Tolls for exposure of animals to sale in Belfast markets, 1845 and 1921

The tolls charged for selling hen’s eggs and poultry are laid out in Schedule E of the Act for the Improvement of the Borough of Belfast, which was enacted in 1845 and remained unchanged until the 1921 Provisional Order for the use of Markets and Weighbridges. Most charges increase over time, but the way in which they were charged also changes. For example, tolls would not be charged for selling fewer than a dozen eggs in 1845 but by 1921, all eggs were subject to tolls, an alteration to which I will return. Suckling pigs also disappear from the list, but “pork, for every carcase” is added, probably a result of the increased regulation of the Belfast Abattoir when it was brought under the control of the Belfast City Council’s Market Committee at the start of the twentieth century (Parkhill and Pollock 2010, p.43). In the graph below, the term “poultry” explicitly includes: “fowls chickens and ducks wild fowls pigeons and rabbits”, which were all charged the same rate for exposure to sale. It is notable that most 1921 tolls are simply doubled from their cost in the 1845 Schedule E (Fig. 12), apart from those on eggs which remain the same and poultry, which are trebled. This may suggest that the value of poultry had increased more relative to other animals and products, but further pricing data would help to clarify this.

### Visibility and Space

From 1877 to 1890, three parcels of Open Space in the westernmost parts of Belfast (Matchett Street, Eastland Street, and Peter’s Hill) were converted to parks and brought under specific legislation, which forbade their use by “goose, duck, or fowl” through bylaws made under the powers of the Open Spaces Acts (PRONI LA/7/1/EB/9). As these species are not named in most other documents of the time which regulate or appropriate public space, it seems likely that those who drafted the legislation expected that poultry would normally be present in the Open Spaces (much like commons) and took pains to exclude them from these areas, thus shaping the animal geography of Belfast at the time. Two decades later, their presence would be further restricted by new bylaws which prevented their sale at all of the dozen markets operating in Belfast at that time, apart from George’s Market on Chichester Street and Ormond Market in Great Patrick Street. Furthermore, it became illegal to pluck poultry in public and a 40 shilling fine would be levied against anyone attempting to do so (PRONI LA/7/1/EB/3). It appears as though poultry may have been left in the markets overnight prior to 1897, as this was also explicitly banned in the carefully-worded legislation and subject to the same 40 shilling fine (PRONI LA/7/1/EB/3). These changes would have further hidden poultry species from the public view and modified the behaviour of people who kept or interacted with them to some extent. These new bylaws do not mention poultry and eggs on the extensive list of goods for which one could be fined for selling at inappropriate times. Finally, a 1965 amendment to the Belfast General Corporation (General Powers) Act (Northern Ireland) of 1961 forbade the sale of live poultry within the city and limited the sale of “plucked poultry” to the Fish Market, with the exception of those sold from approved shops and mobile vans (PRONI LA/7/1/EB/3).

### Commercialisation and the Fancy

After the mid-nineteenth century, poultry products (especially feathers) are omnipresent as a commercial product, as attested to by extensive advertisements for cleaning, dyeing and dressing all manner of feathers for the purposes of fashion (Belfast Morning News, 1 March, 1869a) as well as the sale of items stuffed with chicken and goose feathers (Belfast Morning News, 12 January, 1859a). Along with this increasing commercial visibility came specialty services for re-stuffing and sanitising feather-stuffed items, including the rather dubious claim that a certain Mrs. William MacVeigh of Church Street in Belfast could, by expert cleansing of the feathers from three mattresses, produce enough material to create an additional mattress (Belfast Morning News, 4 February, 1879a). There is also some evidence for the production and sale of equipment for poultry products such as egg frames (Belfast Morning News, 1 April, 1859b). In the Thirtieth Report of the Commissioners of National Education in Ireland for 1863, the list of required items supplied to schools at reduced prices included small brushes, “Camel Hair Pencils, crow-quill, duck-quill, and goose-quill”, the size of which would have been determined by the species of avian the quill was taken from (BPP 1864 XIXPt.II.1, 341, (3351), p.294).

Training and education in poultry-rearing becomes a prominent feature in the documentary record at about the same time. In the 1859d Belfast Morning News for the 5th and 6th of October, an advert for a cook appeared as follows:

“WANTED, a good PLAIN COOK, who can Milk and take charge of Milk and Butter, and who understands the Management of Poultry. A HOUSEMAID also required. Protestants will be preferred. Address by letter “H. J., *Morning News* Office, Belfast.”

Apart from emphasising social divisions, this advertisement suggests that an understanding of the management of poultry was valued and of considerable importance with regard to obtaining gainful employment. Later on, poultry husbandry and cookery were (in addition to dairying) viewed as fundamental aspects of women’s education across Ireland (BPP 1897 XLIII.401,405 (C.8618, C.8619); BPP 1898 XLIV.1, 77, 531 (C.8923, C.8924, C.8925)).

Poultry were also included in the “Annual Cattle Show of the North-East Agricultural Association of Ireland” by 1859 (Belfast Morning News, 1 June, 1859c), when it was held in Belfast with no fee for entry. The rise of the Fancy becomes evident by 1869, when advertisements for the eggs from prize birds of fancy breeds such as the “Black Spanish, Coloured Dorkings, Dark Brahmas, Rouen Ducks, Aylesbury Ducks” and the like appear in the Belfast Morning News (5 March, 1869b). Notice of an auction for other fancy fowl, “Lap-ear’d” rabbits, and guinea pigs is given in the same year (Belfast Morning News, 2 April, 1869c). The inclusion of fancy rabbits and fowl in the same auction is reminiscent of the conventional toll structure set out for poultry and highlights the historical association between breeders of fancy fowl and rabbits (Marie 2008).

Despite not being conventionally thought of as poultry, a pair of “Twelve months old, very handsome” white swans were advertised for sale in the Belfast Morning News by a poultry dealer named John Martin at 120 Botanic Road at the beginning of November, 1869d (3 November). This may reflect a wider trend for fancy pet-keeping and display of unusual animals amongst some classes of society (Thomas 2005). It is unlikely, given the known age of the swans, that they were captured from migratory groups of Bewicke’s or Whooper swans. The Wild Creatures and Forest Laws Act of 1971 consolidated the long-held royal claim to mute swans in what is now Northern Ireland as well as in England and Wales. Prior to this legislation, mute swans and “royal fish” were part of an extensive list of “wild” creatures which were considered to be the domain of the ruling monarch (The Wild Creatures and Forest Laws Act of 1971 C.47 1(1) a.). It is therefore probably unusual that a poultry dealer would secure a pair of them for sale.

### Welfare and Disease

The Belfast branch of the SPCA was founded in 1836 (now the USPCA). In July of 1853, a man was sentenced to a fine of 10s or 14 days in jail for ‘dragging a bag full of ducks through Callender Street in such a manner as to excite the indignation of the passers-by’ (Griffin 1997, p.108 citing the 20 July issue of the Belfast Newsletter). This appears to be the first charge for cruelty against poultry in the Belfast court records, and as Griffin notes, one of only two cases in which public sympathy is aroused (1997, p.108).

The high death rate in Belfast was a matter of serious concern for the local government and inhabitants alike; the Public Health Committee minutes and memoranda from the nineteenth and early twentieth centuries contain various references to this issue and the distress caused by the high prevalence of transmissible disease. The industrial success of Belfast led to increased population densities and pressure upon underdeveloped urban infrastructure (particularly water and sewerage), which worsened the impact of infectious disease (Hassan 1985). Although standardised figures for the city are not available for the entire period, 13–30 % of the 30,000 people living in Belfast from 1819 to 1820 were thought to have suffered from various “fevers”, and the Belfast Fever Hospital reported a patient mortality rate of 13.4 % in 1847–1848 when a particularly deadly strain of typhus was active (Logan 1989, p.84–86). Mass graves such as “plaguey hill” in the Friar’s Bush burial ground were created for the remains of those dying in their thousands from such causes in Belfast (Pheonix 1988; Rugg 2000, p.269). Local notices indicated that deaths often outnumbered births. In 1879 for example, an average of 3 deaths for every birth were advertised in the Belfast Morning News over the course of the year. Tuberculosis was a leading cause of mortality in Belfast. Of the female textile workers who died during 1891–1892, 53 % of them suffered from tuberculosis (Jones 2001, p.70). A series of local legislative measures were designed to tackle infectious disease, including the Belfast Port Sanitary Order (1900), which gave specific rights to prevent spread of contagious disease under the Public Health (Ireland) Acts of 1878 and 1896 and the Infectious Disease Prevention Act (1890). The link between dairy products and tuberculosis was publicly well-established by 1879, when a notice in the Belfast Morning News stated that those involved in milk production must be registered and supply lists of their customers when someone fell ill due to milk-related illness (3 May, 1879). The Dairies, Cowsheds, Milkshops (Ireland) Order of 1908 declared that:

“A person following the trade of Cowkeeper or Dairyman or Purveyor of Milk shall not keep any horses, calves, swine, dogs or poultry in any cowshed or other building used by him for keeping cows, or in any milk store or other place used by him for keeping milk for sale.”

This further demarcated the areas in which poultry could be present, even in an animal husbandry context. Poultry were probably mentioned explicitly due to the recognition by at least 1896 that they were affected by tuberculosis, as testimonies supplied to the Royal Commission on Tuberculosis by a Mr. G.P. Territt and a Dr. R.S. Marsden demonstrate (BPP 1898 XLIX.333, 365 (C.8824, C.8831), p.103, p.328).

### Gender, Education, and Training

Although the practical aspects of poultry husbandry were often considered to be women’s work, engaging in cock-fighting and following the Fancy were predominantly male-associated pastimes (and viewed as hobbies or interests rather than labour or housework). A male interest in poultry was perceived as highly desirable by those wishing to capitalise on the economic potential of the industry, but clearly delineated sociocultural barriers prevented them from fully participating at all levels (as outlined by Bourke 1987). Some men occupied positions of oversight and control, particularly at a national level and in the contexts of training and education, even if they did not interact with poultry on a regular basis.

The Albert National Agricultural Training Institution in Glasnevin (now Albert College) had a very impressive poultry-house constructed by the Board of Public Works which was to be annexed to the Albert Model Farm. This poultry-house was created with the goal of “diffusion of improved breeds of fowls throughout the country”, including “Dorking, Spanish, Bramah Pootra, Creve Coer, Houdan, Toulouse geese, and Aylesbury and Rouen ducks—all of which were originally procured from very eminent breeders in England and Scotland”(BPP 1864 XIXPt.II.1, 341 (3351, 3351-I), p.39). Despite the fact that the consulting expert on this project was a London poulterer by the name of Mr. Baily, it was actually managed by a Mrs. McDonnell (BPP 1864 XIXPt.II.1, 341 (3351, 3351-I), p.39). One particular strand of this diffusion of “improved breeds” in Ireland was the effort by the Congested Districts Board to replace extant types of ‘inferior’ fowl in the northwest of Ireland with larger breeds such as the Minorca and Plymouth Rock from 1892 (BPP 1896 LXVIII.53 (C.8191), p.11). This approach was expensive and problematic, not least because poultry-keepers found the new breeds to be inefficient by their standards. Larger, more quickly maturing chickens did not necessarily translate into immediate production of higher numbers of eggs and as Joanna Bourke has noted, the higher cost of feeding the new breeds of chickens outweighed any increased profit from selling their eggs (Bourke 1987, p.304). The feed consumed by “improved” breeds of chicken literally ate into any profits generated by egg-selling.

Increasingly, poultry husbandry was integrated into national education programmes, and the concept of poultry-keeping as the exclusive domain of women in the context of the household was reinforced by these designs. Despite government claims that poultry husbandry was an important item of the Irish curriculum, only 82 (less than 1 %) of the National Schools in Ireland had small gardens in which gardening and poultry-raising were taught (BPP 1898 XLIV.1, 77, 531 (C.8923, C.8924, C.8923) p.397). In the report of the Commission on Primary Schools under the Board of Education of Ireland, the Principal Teacher at a National School in Enniskerry, Mr. Jeremiah Golden, describes keeping three breeds of chickens in the school garden: Plymouth Rocks, Minorcas, and Orpingtons (BPP 1897 XLIII.401, 405 (C.8618, C.8619) p. 67). When it came to providing details of the provided instruction in poultry-rearing, Mr. Golden stated that his wife undertook that task, rather than himself. The line of questioning inevitably turned to profit and the perception that only “miserable fowl” had been kept before the new, improved breeds were introduced. After the introduction of the “improved breeds” to Enniskerry, chickens would fetch between 1 s 3p each and 2 s at market (roughly the same cost as in Belfast about 50 years previously). In addition to increased profits, the reputation of the area with regard to poultry production had been enhanced by Mr. Golden’s (wife’s) poultry keeping (BPP 1897 XLIII.401, 405 (C.8618, C.8619) p. 67). In the 1898 Schools Final Report, dairying and poultry-keeping are viewed as ideal compulsory subjects for girls at National Schools, with the occasional addition of bee-keeping (BPP 1898 XLIV.1, 77, 531 (C.8923, C8924, C.8925)). Male inspectors of agriculture who trained local teachers did not routinely teach poultry management; this was often reserved for the female teacher only (p.264). At mixed schools where those subjects were incidentally compulsory for boys, the girls were expected to learn, but the “boys won’t become dairymaids” (p.134), implying that the boys were wasting their time by being present at school when they were taught. Although certain agricultural subjects including dairying, poultry management and bee-keeping were thought to have a positive effect on children who might never keep these animals (because it would instil virtues such as thrift and carefulness), it was suggested that children in urban areas, including Belfast, should not be taught them (p.179). One instructor, on being queried regarding the agricultural education of children in the city, responded with an apt quote:

“I taught agriculture in the city for years, and passed children through the results examinations, but they had not an idea of what they were learning; we crammed them as you would cram fowl.” (BPP 1898 XLIV.1, 77, 531 (C.8923, C8924, C.8925) p.143)

**Fig. 13:**
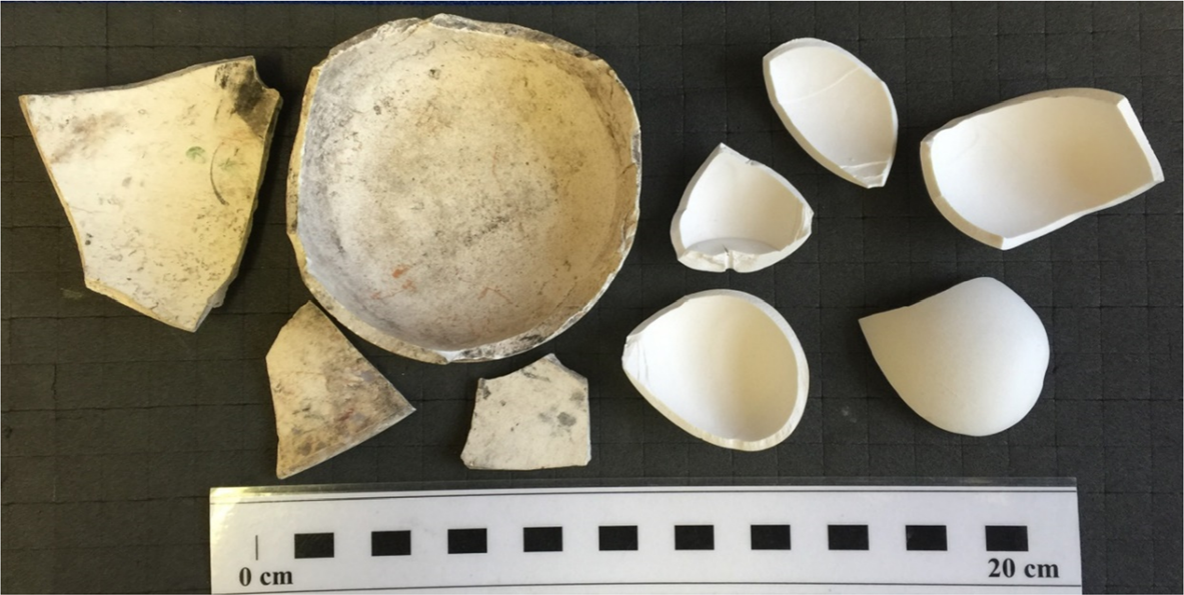
Fragments of a “dummy egg” and eighteenth to nineteenth-century biscuit ware

Cookery instruction was more commonplace, and even small country schools used chicken meat and eggs as fundamental ingredients (BPP 1897 XLIII.401, 405 (C.8618, C.8619) p.141). Amongst other essential life skills routinely taught to children outside of Ireland (such as writing out labels and parcel-tying), egg-packing was considered a desirable inclusion in the Manual and Practical Instruction portion of the Irish curriculum because “it appears very strange to see heavy able-bodied men packing eggs, which is really women’s employment” (BPP 1897 XLIII.401, 405 (C.8618, C.8619) p.90). In the discussion on the mechanics of training children in egg-packing which follows, a potentially useful material correlate for poultry-keeping is alluded to. A manufacturing expert named Mr. Perry suggests that real eggs need not be used for such training as “there are plenty of china eggs” (BPP 1897 XLIII.401, 405 (C.8618, C.8619) p.90). Although it is likely that these “dummy eggs” (used to encourage hens to lay and prevent them from eating eggs) would preserve in the archaeological record, few have been identified in post-medieval assemblages. A notable exception to this is the assemblage from Skipper Street in central Belfast, which contained fragments of china eggs (Ó Baoill 2011 p.121). Depending upon the technique used to produce them (and whether or not they were imported) their fragments could be confused with bone china biscuit ware and they might therefore be overlooked during routine ceramic analyses (Newstead, pers. comm. 2015). Figure 13 below shows fragments of eighteenth to nineteenth-century biscuit ware excavated from a site in Bristol from the teaching collection at the University of Leicester, alongside fragments of a modern “dummy egg”.

Somewhat in contrast to the raising of poultry, which continued to be associated with women in education, training, and elsewhere, there was a gendered transition in the nature of egg-selling in some parts of Ireland over the course of the nineteenth century. In Cootehill (County Cavan), it appears that men became engaged in the work around the time that a centralised market for eggs was established:

“The egg market is of recent date, for 40 years ago women sold them at various points coming into the town. There was no egg market established anywhere at that time, and eggs in large quantities were sold and bought at the various corners and central points… …the men who are engaged in the trade just now object to any change…” (BPP 1889 XXXVIII.1 (C.5888) p.403)

In Dungannon, the group employed in egg-selling were explicitly referred to as “egg men” in discussions of the market accommodation in the town; use of this term suggests that most, if not all, of the individuals involved were men (BPP 1889 XXXVIII.1 (C.5888) p.141).

## Discussion

The archaeological data obtained from the Belfast poultry remains (as well as those data collected from other sites in Ulster and Plymouth) raise interesting questions and offer tantalising possibilities for future research. Certainly, there is a lack of comparative data from the post-medieval period, and bones from avian species are less likely to be analysed than mammalian elements. Still, in contrast to the log-scaled data from chickens excavated from sites in London, the Belfast elements more closely resemble those from other sites in Ulster and sites in Plymouth. This supports the idea that the “best” chickens were sent to the London market, or that the London chickens grew more rapidly (as a result of earlier “improvement”) and were killed at a more consistent age. Alternatively, the element representation could be partly responsible; skeletal elements are expected to differ in varying ways from the “standard chicken” metrics due to their specific anatomical function. Element representation is not consistent across the samples even at the level of appendage: only 38.7 % of the London chicken metrics were taken from wing elements (which tend to be shorter) and 83.3 and 58 % of the elements in the samples from Ulster and Plymouth sites were from the wing. It is also possible that the London elements were from birds which were raised primarily for meat, whilst the Ulster and Plymouth assemblages could contain the remains of dual-purpose or egg-laying birds, which might be expected of small flocks managed at the household level. The presence of very young juveniles in the Belfast assemblage suggests that some poultry were being raised in the city, rather than being universally brought in from the countryside. The age-related arthropathy (joint disease) present in a coracoid supports the interpretation that some chickens were being kept for longer than would be necessary for them to reach maximum weight, which implies an egg-laying purpose. It was not possible to undertake differential diagnosis of the slightly-bowed tibiotarsus due to the fragmentary nature of the element, but the presence of a deficiency-linked condition such as osteomalacia or rickets in urban chickens would be unsurprising, particularly considering the probable nature of their environment, diet, and the existence of “chicken dressers”, furniture which could have further limited the exposure of chickens to daylight. Evidence of dietary deficiency in human remains from 19th-century Kilkenny has been reported (Geber and Murphy 2012) and animal remains have the potential to be similarly revealing. More archaeological data and eggshell analysis, especially from post-medieval assemblages, will be crucial in clarifying the husbandry and health of poultry in past cities.

Egg-selling was a viable way of increasing household income and helping to ensure some financial stability, either by marketing the eggs from one’s own hens or those purchased from another producer. It is important to note that since it cost nothing to sell fewer than a dozen eggs in Belfast from 1845 to 1921, people who couldn’t afford pay a toll could still sell some eggs. This flexibility also extended to hours of exposure for sale (1897). Since poultry and eggs were not included in the list of goods which one could be fined for selling at inappropriate times, their sale could be undertaken outside of market hours without penalty. With regard to the seasonal changes in egg prices becoming less severe between 1825 and 1845, it’s tempting to say that this could be evidence for laying becoming more multi-seasonal over time, though it is difficult to say if this resulted from improvements to husbandry methods. Further in-depth research of the economic data on a yearly basis rather than sampling across decades would help to clarify whether this pattern is an artefact of the available documentary sources rather than a direct reflection of changes in laying productivity. Examination of comparative pricing data on the poultry themselves from other sources over the years 1800–1960 would reveal seasonal trends, show long-term changes in the market value availability of poultry species, illustrate changes in the perception of these species (e.g. when do rabbits stop being priced as “poultry”?) and provide a different angle on the rise of the broiler chicken.

Other reflections of poultry-human relationships are also evident. Cockfighting practices had both Irish and English connections (e.g. fights being held on Ascension Day; using primarily “the odd battle” format) and were carefully arranged not to conflict with each other. They were also held at the same time as horse races, but that relationship did not persist over time. Likewise, the venues at which cockfights take place change over two decades, moving from Ballymoney, Antrim, Carrickfergus, and Newry to locations with smaller populations further south and west. Another interesting aspect of cockfighting in this context is the seasonality of the events. Although it is difficult to trace for other parts of Ireland and Britain, cockfights in America were (and continue to be) held from autumn to summer (Browning 2013). This goes against the presumed seasonality of cockfighting, which should have been limited by feather moulting, during which time the birds could not be fought. However, moulting in chickens can be induced by starvation (Squires 2010, p.179), probably amongst other methods, and it may be that a change in the moulting season was prompted in America but not in the northern regions of Ireland. Many documentary traces of cockfighting disappear long before the practice is banned (in contrast to England), perhaps partly in response to the lack of interest on the part of the wealthy, the lives of whom are more likely to have been recorded.

Welfare is another issue of interest. Investigation of London court cases which featured turkeys showed that they were treated in ways which would now be considered cruel and offensive, but their treatment was never cause for a court case (Fothergill 2014, p.219). It is therefore of considerable interest that a case involving the abuse of ducks was recorded in Belfast; however, the fine for this cruel treatment was 10 shillings, a mere quarter of the fine for plucking poultry in public or leaving one’s chickens in a marketplace overnight according to the 1897 bylaws governing the markets. In Griffon’s history of “the Bulkies”, the early nineteenth-century Belfast police force, a litany of cruel behaviour against animals ends with the assertion that this conduct was commonplace (1997, p.107–108), which makes the public reaction to the abuse of ducks remarkable. Poultry would have been further removed from the public eye through increasing regulation of space in Belfast. These changes would have impacted the habits of poultry-keepers and eventually restricted the husbandry of poultry to homes and nearby exterior spaces. Although the presence of poultry was not of concern to the Public Health Committee (which appears to have dealt with sanitary violations by urban pig-keepers on a regular basis), other experts were aware that poultry species were affected by tuberculosis and may have been concerned that they could transfer the disease to other animals, factors which were surely taken into account when sanitary legislation was developed.

Poultry management was clearly viewed as a desirable skill for women looking to obtain gainful employment, and was a standard feature in the National School curriculum at some schools by the end of the nineteenth century, though agricultural subjects were often optional and perceived as unnecessary in cities, including Belfast. It is possible that urban poultry-keepers gained an understanding about the subject outside of school, and that the transfer of this knowledge and development of related skills took place at a household or community level rather than in a formal classroom context.

Instruction and training in poultry management was heavily gendered across Ireland at various levels. Whereas male Instructors in subjects such as Dairy Management (under which teaching on poultry was often categorised) advised farmers and consulted on agriculture, their female counterparts were expected to take on the practical instruction and were not viewed as experts (BPP 1898 XLIV.1, 77, 531 (C.8923, C8924, C.8925) p.583), despite all evidence to the contrary. Even at the level of a well-resourced National School, male teachers were often not expected to teach poultry husbandry, loath to undertake instruction of the subject, and some left it to their wives (BPP 1897 XLIII.401, 405 (C.8618, C.8619) p.75). It is little wonder that men could not be encouraged to take on even the more prominent poultry-related roles in the face of such deeply-entrenched gendering of the practice in both Ireland and Britain (Bourke (1987, 1993) and Sayer 2013).

Poultry species were present in Belfast for centuries before the industrial expansion of the city in the later post-medieval period, and they occupied a variety of roles. Regardless of whether these animals were cock-fighting, acting as a focus of seasonal feasting (and thieving), producing eggs (for use, sale, or Eastertime trundling), or being plucked for fashion, mattress-stuffing, or brush-quills, these species featured largely in the lived environment of urban Belfast. They were also viewed as possible hosts for disease, and their presence and treatment was considered and regulated, despite their low archaeological and archival visibility in comparison to species which stand out because they are viewed as more companionable or dangerous (dogs) or less hygienic (pigs). Poultry were only a minor aspect of the later post-medieval urban economy in Ireland and Britain, and their husbandry in cities is all but uncharted. However, as observed by Short with regard to the smallholders in the Weald of Sussex (1982), keeping them could help people with limited incomes to survive short-term difficulties, and probably provided a form of food security. Despite the limitations of analysed material, the archaeological and documentary evidence suggests that the inhabitants of St. Anne’s Square and other Belfast neighbourhoods were keeping chickens, perhaps for small-scale egg production. At least some hens were kept into advanced age, and a reduction in seasonal egg price variation during the mid-nineteenth century could more generally indicate improved, more multi-seasonal egg-laying. The toll and market structure of later post-medieval Belfast would have permitted egg-selling to generate a small but reliable source of income until around 1921, when Schedule E was revised. The husbandry methods used in Belfast and surrounding areas would have been altered over time in response to legislation on the management of increasingly urban spaces, evolving perspectives on hygiene (especially with regard to transmissible disease), and changing views on the public appropriateness of certain poultry-related activities. Further publication of archaeological reports and faunal analyses from the post-medieval period, combined with in-depth historical research, would greatly enhance our understanding of human-animal relationships in urban environments.

## References

[CR1] Advertisement, May 14th. (1765a). *Belfast Newsletter and General Advertiser*. PRONI MIC19/14.

[CR2] Advertisement, May 28th. (1765b). *Belfast Newsletter and General Advertiser*. PRONI MIC19/14.

[CR3] Advertisement, June 14th. (1765c). *Belfast Newsletter and General Advertiser*. PRONI MIC19/14.

[CR4] Advertisement, June 25th. (1765d). *Belfast Newsletter and General Advertiser*. PRONI MIC19/14.

[CR5] Advertisement, 15–19 April. (1785a). *Belfast Newsletter*. PRONI MIC19/34.

[CR6] Advertisement, 24–27 May. (1785b). *Belfast Newsletter*. PRONI MIC19/34.

[CR7] Advertisement, May 27–31. (1785c). *Belfast Newsletter*. PRONI MIC19/34.

[CR8] Advertisement, yearly egg prices. (1825). *Belfast Newsletter*. PRONI MIC19/73.

[CR9] Advertisement, yearly egg prices. (1845a). *Belfast Newsletter*. PRONI MIC19/92.

[CR10] Advertisement, yearly poultry prices. (1845b). *Belfast Newsletter*. PRONI MIC19/92.

[CR11] Advertisement, 12 January. (1859a). *Belfast Morning News*. PRONI MIC296/3.

[CR12] Advertisement, 1 April. (1859b). *Belfast Morning News*. PRONI MIC296/3.

[CR13] Advertisement, 1 June. (1859c). *Belfast Morning News*. PRONI MIC296.

[CR14] Advertisement, 5th and 6th of October. (1859d). *Belfast Morning News*. PRONI MIC296.

[CR15] Advertisement, 1 March. (1869a). *Belfast Morning News*. PRONI MIC296/32.

[CR16] Advertisement, 5 March. (1869b). *Belfast Morning News*. PRONI MIC296/32.

[CR17] Advertisement, 2 April. (1869c). *Belfast Morning News*. PRONI MIC296/32.

[CR18] Advertisement, 3 November. (1869d). *Belfast Morning News*. PRONI MIC296/32.

[CR19] Advertisement, 4 February. (1879a). *Belfast Morning News*. PRONI MIC296/50.

[CR20] Advertisement, 3 May. (1879b). *Belfast Morning News*. PRONI MIC296/50.

[CR21] Allan J, Barber J, Gaimster D, Redknap M (1992). A seventeenth-century pottery group from Kitto. Everyday and Exotic.

[CR22] An Act for the Improvement of Belfast. (1845). PRONI LA/7/1/B/2.

[CR23] Announcement, February 23rd. (1749). *Belfast Newsletter.* PRONI MIC/19/4 23.

[CR24] Announcement, yearly births and deaths. (1879). *Belfast Morning News*. PRONI MIC296/49.

[CR25] Atkinson, H. (1891). The old english game fowl: its history, description, management, breeding and feeding. *Fanciers’ Gazette*.

[CR26] Bardon J (1982). Belfast: an illustrated history.

[CR27] Bartlett, C. M. (2014). *A history of newspaper journalism in Belfast, 1855–1910* (Doctoral dissertation, Ulster University).

[CR28] Beeton I (1861). The Book of Household Management.

[CR29] Belfast General Corporation (General Powers) Act (Northern Ireland). (1961). PRONI LA/7/1/EB/3.

[CR30] Belfast Port Sanitary Order. (1900). PRONI LA/7/1/B/2.

[CR31] Bourke, J. (1987). Women and Poultry in Ireland, 1891–1914. *Irish Historical Studies* (May): 293–310.

[CR32] Bourke J (1993). Husbandry to housewifery: women, economic change, and housework in Ireland, 1890–1914.

[CR33] Brannon N (1985). Archaeological Excavations at Dungiven Priory and Bawn. Benhradagh.

[CR34] Brannon N, Hamlin A, Lynn C (1988). Belfast. Pieces of the Past: Archaeological excavations by the Department of the Environment for Northern Ireland 1970–1986.

[CR35] Brannon, N. (1989). Unpublished report on Bellaghy Bawn.

[CR36] Brewster MP, Reyes CL (2013). Animal Cruelty.

[CR37] Browning, W. (2013). The Last Cockfighter Tells All. *SB Nation*. Available at http://www.sbnation.com/longform/2013/1/16/3880340/the-last-cockfighter-tells-all accessed 30/11/2015.

[CR38] Bye-Laws for the better Regulation of the Markets of the Borough of Belfast. (1897). PRONI LA/7/1/EB/3.

[CR39] Cohen A, Serjeantson D (1996). A Manual for the Identification of Bird Bones from Archaeological Sites.

[CR40] Collins T, Martin J, Vamplew W (2005). Encyclopedia of Traditional British Rural Sports.

[CR41] Commissioners of National Education in Ireland. The thirtieth report of the Commissioners of National Education in Ireland, (1863). With appendices. Vol. I. 1864 (3351) (3351-I) XIX Pt.II.1, 341. Available at http://gateway.proquest.com/openurl?url_ver=Z39.88-2004&res_dat=xri:hcpp&rft_dat=xri:hcpp:rec:1864–040140 accessed 30/11/2015.

[CR42] Conder, T., and Hogg, A. (1780). *A new & correct map of the Province of Ulster, Drawn from the Latest & Best Authorities.*

[CR43] Congested Districts Board for Ireland. Fifth report of the Congested Districts Board for Ireland, for the period from the 1st of April, 1895, to the 31st of March, 1896. 1896 (C.8191) LXVII.53. Available at http://gateway.proquest.com/openurl?url_ver=Z39.88-2004&res_dat=xri:hcpp&rft_dat=xri:hcpp:rec:1896–074145 accessed 30/11/2015.

[CR44] Connolly SJ (2012). Belfast 400: People, place and history.

[CR45] Crown Book at Quarter Session – Antrim. (1822–1825). PRONI ANT/1/1/A/1.

[CR46] Crown Book at Quarter Session – Antrim. (1847). PRONI ANT/1/1/A/3.

[CR47] Crown Book at Quarter Session – Belfast. (1902–1913). PRONI ANT/1/1/A/9.

[CR48] Cutter R (1989). Brocade and blood: the cockfight in Chinese and English poetry. Journal of the American Oriental Society.

[CR49] Denham SD, Dunlop C (2008). Appendix 12: Animal Bone. Archaeological Excavation Report: St. Anne’s Square, Belfast.

[CR50] Donlon J (1990). Fighting cocks, feathered warriors, and little heroes. Play & Culture.

[CR51] Dunlop C (2008). Archaeological Excavation Report: St. Anne’s Square, Belfast.

[CR52] Forsyth I (1978). The theme of cockfighting in Burgundian Romanesque sculpture. Speculum.

[CR53] Fothergill BT (2014). The husbandry, perception and ‘improvement’ of turkeys in Britain, 1500–1900. Post-Medieval Archaeology.

[CR54] Geber J, Murphy E (2012). Scurvy in the Great Irish Famine: Evidence of Vitamin C Deficiency From a Mid-19th Century Skeletal Population. American Journal of Physical Anthropology.

[CR55] Griffin B (1997). The bulkies: police and crime in Belfast, 1800–1865.

[CR56] Hamlin, A., and Lynn, C. (1988). Pieces of the Past: Archaeological excavations by the Department of the Environment for Northern Ireland 1970–1986. Stationery Office Books, Belfast.

[CR57] Hassan JA (1985). The Growth and Impact of the British Water Industry in the Nineteenth Century. The Economic History Review.

[CR58] Horning A (2006). Focus found. New directions for Irish historical archaeology. Archaeological Dialogues.

[CR59] Infectious Disease Prevention Act. (1890). PRONI LA/7/1/B/2.

[CR60] Jones G (2001). Captain of these men of death”: the history of tuberculosis in nineteenth and twentieth century Ireland.

[CR61] Logan, J. S. (1989). Trench fever in Belfast, and the nature of the ‘relapsing fevers’ in the United Kingdom in the nineteenth century. *The Ulster Medical Journal 58*(1): 83–88. PMC2448553 2672525

[CR62] Maguire W (2009). Belfast: A History.

[CR63] Marie J (2008). For Science, Love and Money: The Social Worlds of Poultry and Rabbit Breeding in Britain, 1900—1940. Social Studies of Science.

[CR64] Mason WS (1819). A statistical account, or parochial survey of Ireland (Vol.

[CR65] Maxwell, Herbert Eustace, Sir. Royal commission on tuberculosis. Report of the Royal commission appointed to inquire into the administrative procedures for controlling danger to man through the use as food of the meat and milk of tuberculous animals. Part I. Report. 1898 (C8824) (C.8831) XLIX.333, 365. Available at http://gateway.proquest.com/openurl?url_ver=Z39.88-2004&res_dat=xri:hcpp&rft_dat=xri:hcpp:rec:1898–075992 accessed 30/11/2015.

[CR66] Moss MS (1986). Shipbuilders to the world: 125 years of Harland and Wolff, Belfast 1861–1986.

[CR67] Ó Baoill R (2011). Hidden history below our feet: The archaeological story of Belfast.

[CR68] O’Connor T (2008). The archaeology of animal bones.

[CR69] O’Hara P, van der Burg M, Endeveld M (1994). Out of the shadows: Women on family farms and their contribution to agriculture and rural development. Women on family farms: Gender research, EC policies and new perspectives.

[CR70] Owen DJ (1921). History of Belfast.

[CR71] Parkhill T, Pollock V (2010). Belfast: Events, People and Places over the 20th Century.

[CR72] Pheonix E (1988). Two acres of Irish history: a study through time of Friar’s Bush and Belfast 1570–1918.

[CR73] Philo C (1995). Animals, geography, and the city: notes on inclusions and exclusions. Environment and Planning D: Society and Space.

[CR74] Public Health (Ireland) Act. (1878, 1896). PRONI LA/7/1/B/2.

[CR75] Rugg J (2000). Defining a place of burial: What makes a cemetery a cemetery?. Mortality.

[CR76] Sayer K (2013). His footmarks on her shoulders’: the place of women within poultry keeping in the British countryside, c. 1880 to c. 1980. Agricultural History Review.

[CR77] Scott G (1957). The History of Cockfighting.

[CR78] Secord J (1981). Nature’s fancy: Charles Darwin and the breeding of pigeons. Isis.

[CR79] Sentencing, 20 July. (1853). *Belfast Newsletter*. (Cited in Griffin, B. (1997). The bulkies: police and crime in Belfast, 1800–1865. Irish Academic Press, Sallins.)

[CR80] Short B (1982). The Art and Craft of Chicken Cramming’: Poultry in the Weald of Sussex 1850–1950. The Agricultural History Review.

[CR81] Sketchley, W. (1814). *The Cocker; containing Every Information to the Breeders and Amateurs of the noble bird, The Game Cock: to which is added, a variety of other useful information for The Instruction of those who are attendants on the Cock Pit.* J. Croft, London.

[CR82] Squires EJ (2010). Applied Animal Endocrinology.

[CR83] Stanley, Edward Henry, 15th Earl of Derby. Royal Commission on Market Rights and Tolls. Reports of Mr. Charles W. Black, assistant commissioner, together with the minutes of evidence taken in the province of Ulster, and portions of the provinces of Leinster and Connaught. Vol. V. 1889 (C.5888) XXXVIII.1. Available at http://gateway.proquest.com/openurl?url_ver=Z39.88-2004&res_dat=xri:hcpp&rft_dat=xri:hcpp:rec:1889–065714 accessed 30/11/2015.

[CR84] Stead P (2003). Excavation of the Medieval and Later Waterfront at Dung Quay, Plymouth. Proceedings of the Devon Archaeological Society.

[CR85] Strutt J (1801). The sports and pastimes of the people of England: including the rural and domestic recreations, May games, mummeries, shows, processions, pageants, and pompous spectacles, from the earliest period to the present time.

[CR86] The Dairies, Cowsheds, Milkshops (Ireland) Order. (1908). PRONI LA/7/1/EB/27.

[CR87] Thirsk J (1997). Alternative Agriculture: A History From the Black Death to the Present Day.

[CR88] Thirsk J (2006). Food in early modern England: phases, fads, fashions 1500–1760.

[CR89] Thomas R, Plukowski A (2005). Perceptions versus reality: changing attitudes towards pets in medieval and post-medieval England. Just Skin and Bones? New Perspectives on Human-Animal Relations in the Historic Past, BAR International Series 1410.

[CR90] Thomas R (2009). Bones of contention: why later post-medieval assemblages of animal bones matter. Crossing Paths or Sharing Tracks: Future Directions in the Archaeological Study of Post-1550 Britain and Ireland.

[CR91] Thomas RH, Holmes M, Morris J (2013). So bigge as bigge may be”: tracking size and shape change in domestic livestock in London (AD 1220–1900). Journal of Archaeological Science.

[CR92] Vann, S., and Thomas, R. (2006). Humans, other animals and disease: a comparative approach towards the development of a standardised recording protocol for animal palaeopathology. *Internet Archaeology* 20.

[CR93] Verdon N (2009). Agricultural Labour and the Contested Nature of Women’s Work in Interwar England and Wales. The Historical Journal.

[CR94] Walsh, William J. Commission on manual and practical instruction in primary schools under the Board of National Education in Ireland. Final report of the commissioners. 1898 (C.8923) (C.8924) (C.8925) XLIV.1, 77, 531. Available at http://gateway.proquest.com/openurl?url_ver=Z39.88-2004&res_dat=xri:hcpp&rft_dat=xri:hcpp:rec:1898–075975 accessed 30/11/2015.

[CR95] Walsh, William J. Commission on manual and practical instruction in primary schools under the Board of National Education in Ireland. Third report of the commissioners. 1897 (C.8618) (C.8619) XLIII.401, 405. Available at http://gateway.proquest.com/openurl?url_ver=Z39.88-2004&res_dat=xri:hcpp&rft_dat=xri:hcpp:rec:1897–074879 accessed 30/11/2015.

